# Stress, Hope, and Depression Among Older Adults Living in Public Housing

**DOI:** 10.1093/geroni/igaa057.1084

**Published:** 2020-12-16

**Authors:** Mignon Montpetit, LaKeesha James-Smith, Amy Gourley

**Affiliations:** 1 Illinois Wesleyan University, Bloomington, Illinois, United States; 2 Bloomington Housing Authority, Bloomington, Illinois, United States; 3 Illinois Wesleyan University, Carlock, Illinois, United States

## Abstract

Individuals living in public housing often experience myriad stressors related to poverty and mental illness. The current study explores how hope impacts the relationship between stress and depression in a sample of adults (aged 51-90 years; Mage= 63.3 years; SDage= 8.6 years) living in public housing. Questionnaire data were collected before and after running an intervention geared toward improving residents’ well-being. Results of the initial questionnaire study suggest that hope moderates the stress -> depression relationship (p = .001), with effects in the expected directions: individuals exhibiting higher-than-average levels of stress and below-average hope reported the highest levels of depression. Data further suggest modest increases in hope post-intervention (p = .06). Overall, results suggest that hope may be important in helping mitigate the impact of life stress on vulnerable individuals, and that it can be augmented in the context of a short-term, cost-effective intervention.

